# Optimizing Dual‐Plate Fixation of Distal Femoral Periprosthetic Fractures Using a Distal Tibial Metaphyseal Plate

**DOI:** 10.1111/os.70420

**Published:** 2026-08-31

**Authors:** Che Lin, Yueh‐Ying Hsieh

**Affiliations:** ^1^ Department of Orthopedics, Shuang Ho Hospital Taipei Medical University New Taipei Taiwan; ^2^ Department of Orthopaedics, School of Medicine, College of Medicine Taipei Medical University Taipei Taiwan; ^3^ Department of Orthopedics, Hsin Kuo Min Hospital Taipei Medical University Taoyuan City Taiwan

**Keywords:** distal femoral periprosthetic fractures, distal tibial metaphyseal plate, dual‐plate fixation

## Abstract

**Introduction:**

Distal femoral periprosthetic fractures following total knee arthroplasty (TKA) are complex and technically demanding injuries in orthopedic trauma surgery. With the increasing prevalence of TKA in an aging population, the incidence of these fractures has risen steadily. This study aims to share our experience of implant selection and surgical strategy, which provides an alternative and out‐of‐the‐box thinking process for fracture fixation.

**Methods:**

Between 2022 and 2024, 10 consecutive patients with distal femoral periprosthetic fractures involving a well‐fixed femoral component were treated using a dual‐plate construct incorporating a distal tibial metaphyseal locking plate for medial augmentation and distal femoral plate. Clinical, radiographic, and functional outcomes were retrospectively collected and analyzed.

**Results:**

All patients were followed for a minimum of 12 months. Radiographic callus formation was observed at approximately 2 months postoperatively, and fracture union was achieved at a mean of 4 months. No cases of fixation failure, nonunion, or deep infection were observed during the follow‐up period. Favorable functional outcomes were also noted at final follow‐up.

**Conclusion:**

The use of a dual‐plating construct that may provide enhanced mechanical stability was well established, allowing early mobilization and progression to weight‐bearing. The distal tibial metaphyseal plate, which even though was not designed original for treating such kind of fracture, demonstrated good anatomical conformity to the medial distal femur and may serve as a practical option for medial augmentation. Orthopedic surgeons should not be constrained by the designation of a plate as an “anatomical plate”; rather, they should make judicious use of the implants available and apply them in the manner most appropriate for the specific fracture pattern. Using distal tibial metaphyseal plate as medial augmentation in dual‐plate fixation appears to be a feasible and promising strategy for managing distal femoral periprosthetic fractures following TKA.

## Graphical abstract

This study presents the preliminary evaluation of the clinical, radiographic, and functional outcomes of dual‐plating technique for distal femoral periprosthetic fractures. To our knowledge, this is the first clinical report directly using a distal tibial metaphyseal plate for the dual‐plating technique, highlighting its potential advantages in curvature fitness and structural stability.

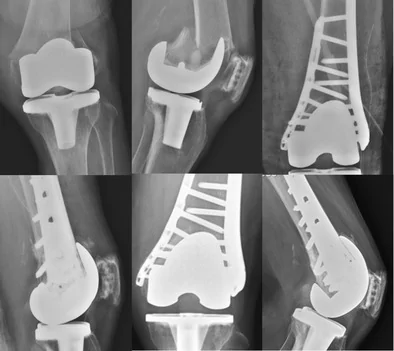

## 1 Introduction

As global life expectancy continues to rise and functional expectations increase, the number of patients undergoing total knee arthroplasty (TKA) has grown substantially. In recent years, more than 790,000 TKAs have been performed annually in the United States alone [1]. With the expanding population of patients living with knee prostheses, distal femoral periprosthetic fractures have become increasingly prevalent, particularly following low‐energy falls in elderly individuals [2, 3]. Recent systematic reviews report an incidence of periprosthetic fractures after TKA ranging from 0.3% to 2.5%, with the majority occurring in the distal femur [3].

The management of distal femoral periprosthetic fractures remains challenging, and the surgical principles differ from those applied to native distal femoral fractures [4, 5, 6, 7]. In addition to the fracture pattern, surgeons must address potential interference from the femoral component, implant loosening, compromised bone stock, and severe osteoporosis [3, 8, 9, 10, 11, 12]. Achieving an appropriate balance between construct stability and biological preservation is critical, as excessively rigid fixation may impair secondary bone healing, whereas insufficient stability may result in delayed union or nonunion [4].

Currently available surgical options include lateral locking plate fixation, retrograde intramedullary nailing, and dual‐plating constructs [13]. However, the mechanical reliability of single lateral plating has been questioned, particularly in the absence of medial column support, which may predispose to varus collapse and fixation failure [14, 15, 16, 17, 18]. Retrograde intramedullary nailing, although minimally invasive, is often limited by femoral component design, distal femoral anatomy, and femoral bowing, particularly in elderly Asian populations. A 101 year‐old woman with previous right TKA surgery has undergone a falling down episode with right thigh contusion. At that time, DePuy Synthes retrograde nail was utilized during the open reduction and internal fixation surgery for her right distal femoral periprosthetic fracture (Figure 1). These limitations have led to increasing interest in dual‐plating strategies to enhance construct stability (Table 1) [10, 19, 20, 21, 22, 23].

**FIGURE 1:**
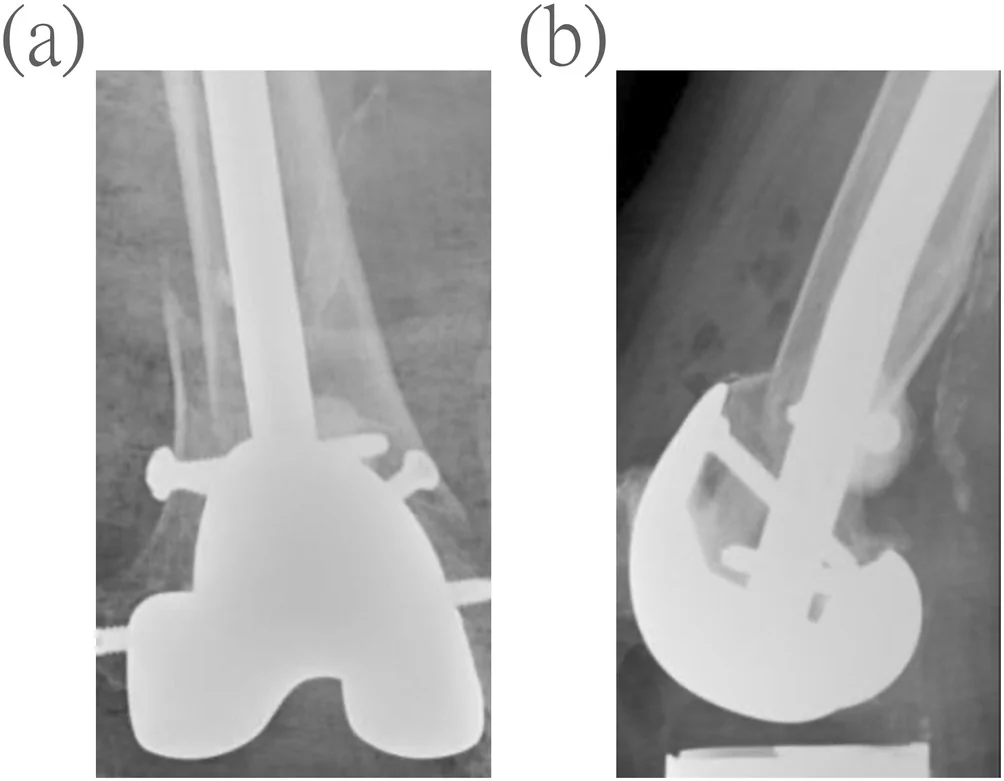
(a) and (b) A 101‐year‐old woman with a right distal femoral periprosthetic fracture who underwent open reduction and internal fixation with a DePuy Synthes retrograde nail.

**TABLE 1:** Advantages and disadvantages of the available surgical options for distal femoral periprosthetic fracture fixation.

Surgical option	Lateral plating	Retrograde nailing	Dual plating
Advantages	Traditionally used technique with a unilateral incision and shorter operative time	Promotes secondary healing without an extensile surgical incision	Improved construct stability Consistent with the triangle‐of‐stability concept in the distal humerus
Disadvantages	Lacks medial buttress support May result in late varus deformity Distal screw purchase may be limited by the existing femoral component	Femoral component interference Restriction related to nail bowing	Longer operative time More extensive surgical exposure

Dual plating, conceptually analogous to the “triangle of stability” in distal humerus fixation, has been increasingly applied to distal femoral periprosthetic fractures (Figure 2) [24, 25]. Previous biomechanical and clinical studies have suggested that dual‐plating constructs may provide improved stability and favorable outcomes compared with single‐plate fixation. However, optimal implant selection for medial augmentation remains unclear, and a precontoured anatomic plate specifically designed for the medial distal femur is not currently available. Commonly used implants, such as straight locking compression plates or locking reconstruction plates, often require intraoperative contouring, which may result in suboptimal plate‐to‐bone conformity and limited screw trajectory options.

**FIGURE 2:**
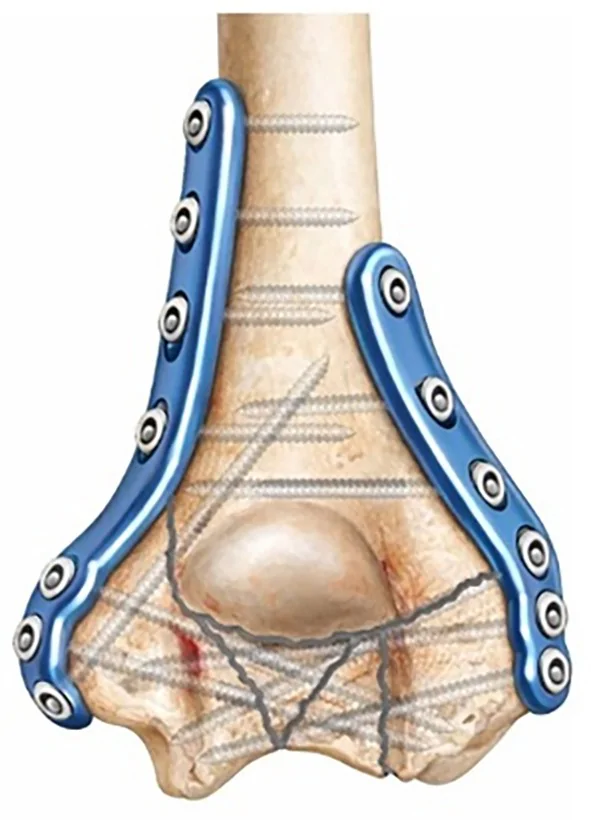
The triangle‐of‐stability concept in distal humerus fixation [24, 25], applied here to distal femoral periprosthetic fracture fixation.

To address this issue, we evaluated several commercialized anatomical locking plates and observed that the distal tibial metaphyseal plate demonstrates favorable conformity to the medial curvature of the distal femur and allows versatile screw placement. Based on this observation, we adopted a dual‐plate construct using a lateral Less Invasive Stabilization System (LISS) plate in combination with a medial distal tibial metaphyseal plate.

The purpose of this study was as follows:

1. to investigate the feasibility of using a distal tibial metaphyseal plate as an alternative medial fixation device for the treatment of distal femoral periprosthetic fractures, extending its application beyond its conventional anatomical indication; and
2. to evaluate the clinical, radiographic, and functional outcomes of this novel dual‐plating strategy in a consecutive series of patients, with particular emphasis on its potential clinical applicability.

## 2 Materials and Methods

### 2.1 Patient Selection

Between January 2022 and December 2024, we retrospectively reviewed 10 consecutive patients who sustained distal femoral periprosthetic fractures following total knee arthroplasty (TKA) and were surgically treated at a single institution. Consecutive enrollment was used to minimize selection bias.

Inclusion criteria were a history of primary TKA, a fracture line located just proximal to or within 2 cm of the femoral component, a well‐fixed femoral implant, and preserved preinjury ambulatory function with independence in activities of daily living. Implant stability was assessed preoperatively using radiographic evaluation, including the absence of radiolucent lines, component subsidence, or malalignment. Computed tomography (CT) was used in selected cases to further evaluate the bone–implant interface. Exclusion criteria in our research includes acute infection such as osteomyelitis, pathological fracture, periprosthetic fracture with loosening femoral component, and joint contracture caused by prolonged bedridden.

Intraoperatively, femoral component stability was routinely confirmed by direct visualization and manual stress testing after exposure of the distal femur. Patients with suspected or confirmed femoral component loosening, active periprosthetic joint infection, long‐term bedridden status, or impaired consciousness were excluded, as these conditions would require alternative surgical strategies such as revision arthroplasty.

All enrolled patients were women, with a mean age of 80.0 years (range, 63–89 years), reflecting the typical demographic profile of osteoporotic geriatric patients sustaining low‐energy periprosthetic fractures. All patients were treated with open reduction and internal fixation (ORIF) using a dual‐plating technique. Demographic data and baseline characteristics are summarized in Table 2.

**TABLE 2:** Clinical characteristics, perioperative variables, and outcomes of the 10 included patients.

Patient	Case 1	Case 2	Case 3	Case 4	Case 5	Case 6	Case 7	Case 8	Case 9	Case 10
Sex	Female	Female	Female	Female	Female	Female	Female	Female	Female	Female
Side	Right	Left	Left	Right	Left	Right	Right	Left	Left	Left
Age (years)	81	81	89	85	63	85	83	85	80	66
Time since primary TKA (years)	10	4	10	13	10	10	5	12	8	12
Comorbidity	Stroke	Hypertension Gout	Hypertension CAD Hyperlipidemia	Hypertension DM ESRD Stroke Dementia	No underlying diseases	Hypertension	No underlying diseases	CAD Hyperlipidemia	Hypertension DM	Hypertension DM ESRD
Operative time (min)	156	154	146	215	191	170	150	160	185	185
Blood loss (mL)	800	500	500	750	750	800	1000	1000	750	750
Intraoperative blood transfusion (mL)	1000	500	1000	500	500	500	1000	1000	500	500
Follow‐up (months)	15	15	18	12	12	12	15	18	15	12
Bony union (months)	3	3	3	5	3	3	3	3	3	3
BMD T‐score	−2.8	−3.7	−3.6	−4.0	−3.0	−3.3	−3.5	−3.8	−3.7	−3.2
Postoperative ROM	0°–120° near full	0°–90°	0°–120° near full	0°–120° near full	0°–120° near full	0°–120° near full	0°–90°	0°–120° near full	0°–120° near full	0°–120° near full
Time to start weight‐bearing (weeks)	6	8	6	6	6	6	6	8	6	6
KOOS score	88	92	95	83	81	90	87	89	92	91
VAS score	2	1	1	3	2	1	1	2	1	3

Abbreviations: BMD: bone mineral density; CAD: coronary artery disease; DM: diabetes mellitus; ESRD: end‐stage renal disease; KOOS: Knee Injury and Osteoarthritis Outcome Score; ROM: range of motion; TKA: total knee arthroplasty; VAS: visual analogue scale.

### 2.2 Clinical and Radiographic Evaluations

Demographic and perioperative variables were collected, including age, fracture laterality, time interval from index TKA to fracture, comorbidities, operative time, intraoperative blood loss, and transfusion volume. Postoperative bone mineral density (BMD) was assessed using dual‐energy X‐ray absorptiometry to characterize systemic bone quality.

Preoperative evaluation included standardized anteroposterior and lateral radiographs and CT scans to define fracture morphology and assess implant stability. Postoperative radiographs were obtained immediately after surgery and at 1, 3, 6, and 12 months to evaluate reduction quality, implant position, and fracture healing. CT was performed at approximately 6 months postoperatively to confirm bridging callus formation when necessary.

Patients were followed at regular intervals (2 weeks, 1, 2, 3, 6, and 12 months postoperatively, and every 3 months thereafter). The mean follow‐up duration was 15 months (range, 12–18 months).

Clinical outcomes included knee range of motion (ROM), time to initiation of weight‐bearing, Visual Analog Scale (VAS) pain score, and Knee Injury and Osteoarthritis Outcome Score (KOOS), assessed at 1, 3, 6, and 12 months postoperatively.

Nonunion was defined as the absence of radiographic progression toward healing at 9 months postoperatively or no interval progression over 3 consecutive months. Malunion was defined as coronal deformity > 5°, sagittal deformity > 10°, rotational deformity > 15° (assessed on CT), or limb‐length discrepancy > 2 cm.

Given the small sample size, only descriptive statistics were performed.

### 2.3 Surgical Technique and Implant Selection

In our study, we utilized DePuy distal tibial metaphyseal plate for medial fixation. According to one previous study, medial plating of distal femur fracture with inappropriate plate position and improper contour could eventually lead to varus deformity, the so‐called “golf club deformity” [26]. However, the DePuy distal tibial metaphyseal plate, originally designed for tibia metaphyseal fracture, conforms closely to the medial curvature of distal femur. It provides multiple fixation points with versatile screw trajectories and maintains intimate plate‐to‐bone contact (Figure 3A,B). This is particularly valuable in patients with severe osteoporosis and in the presence of a previous TKA femoral component, where screw placement options may be limited when choosing conventional LCP. The relatively low‐profile design of this plate also seems to decrease soft tissue irritation and improve postoperative knee range of motion.

**FIGURE 3:**
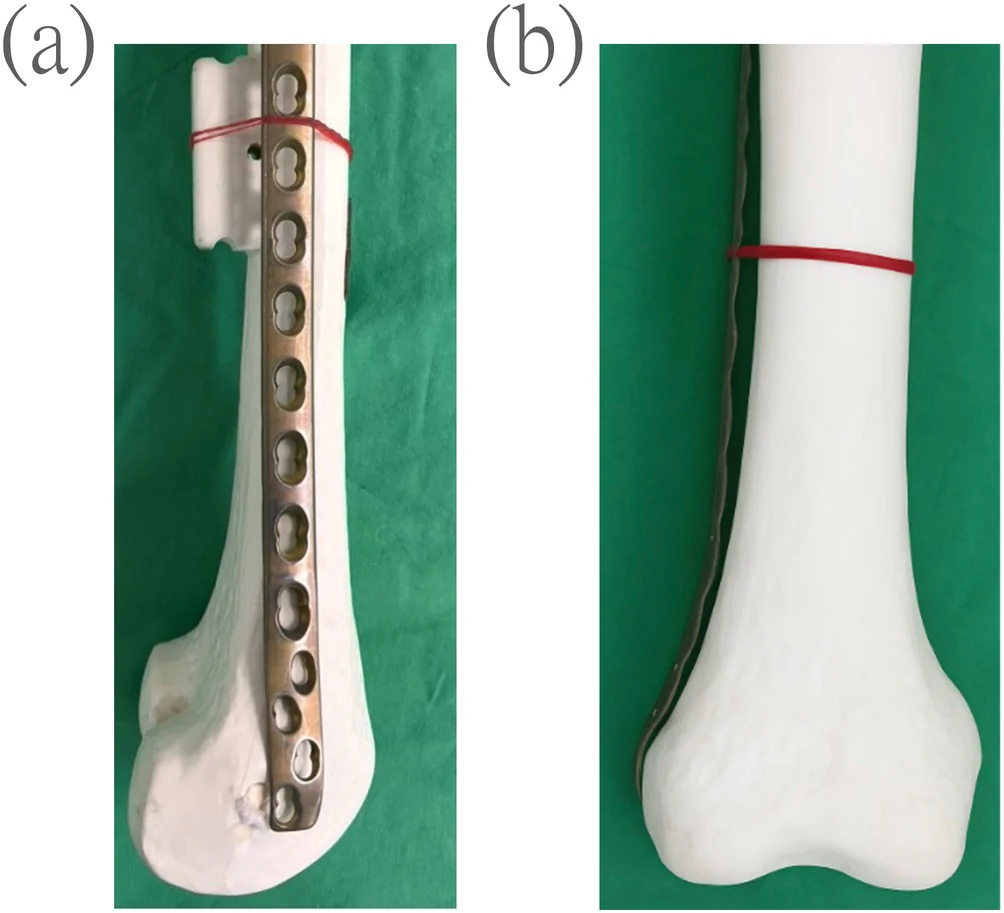
(a) and (b) The DePuy distal tibial metaphyseal plate closely matches the curvature of the medial distal femur and appears to be an ideal implant for providing sufficient medial buttress support in distal femoral periprosthetic fractures.

During surgery all patients were positioned supine on a radiolucent operating table, and all procedures were performed under continuous fluoroscopic guidance, following principles analogous to those applied in the management of isolated distal femoral fractures. No tourniquet was used in any case to avoid potential surgical site contamination related to the proximity of the tourniquet device to the operative field. A dual‐incision approach was used, with longitudinal incisions placed parallel to the medial and lateral borders of the distal femur to allow direct fracture visualization and optimal plate positioning.

Laterally, the skin incision was initiated along the mid‐lateral line of the femoral shaft at the level of Gerdy's tubercle and curved proximally over the lateral femoral condyle. After division of the subcutaneous tissue, the iliotibial band was incised in line with the skin incision. In the distal 8–10 cm of the femur, the muscle fibers of the vastus lateralis are minimal. The fascia surrounding the vastus lateralis was incised just anterior to the lateral intermuscular septum, and the muscle fibers were carefully elevated off the septum from distal to proximal. The vastus lateralis was then retracted anteromedially to expose the lateral aspect of the distal femur and the fracture site.

Medially, a second longitudinal incision was made along the course of the adductor magnus tendon. The adductor tubercle was identified, and the tendon trajectory was marked proximally. A straight incision was made along the posterior border of the adductor magnus tendon. After deep dissection, the anterior border of the sartorius muscle was identified and retracted posteriorly, exposing the adductor magnus tendon. The adductor magnus muscle and tendon were subsequently retracted posteriorly, while the vastus medialis was retracted anteriorly, allowing direct visualization of the medial aspect of the distal femur.

Anatomic reduction was achieved under direct visualization and confirmed by fluoroscopic imaging. Provisional stabilization was obtained using Kirschner wires. Fixation was performed sequentially, beginning on the lateral side. A DePuy Less Invasive Stabilization System plate was applied to the lateral distal femur through the lateral incision, with stepwise insertion of locking screws to secure the construct. Subsequently, a DePuy distal tibial metaphyseal locking plate was positioned through the medial incision to provide medial buttress support, thereby enhancing overall biomechanical stability. At least four proximal locking screws were placed on each side of the femur to ensure adequate fixation length and optimal load distribution.

Final reduction, plate position, and screw placement were meticulously confirmed under fluoroscopic guidance. The wounds were thoroughly irrigated, and the muscle fascia and soft tissues were closed in layers. A long‐leg splint was applied postoperatively to protect the fixation construct during the early healing phase.

### 2.4 Rehabilitation Protocol

Postoperatively, all patients were managed according to a standardized rehabilitation protocol tailored for geriatric periprosthetic fracture care. Continuous passive motion was initiated immediately after surgery to facilitate early knee mobility and minimize postoperative stiffness. Emphasis was placed on early range‐of‐motion exercises while carefully protecting the fixation construct. Given the osteoporotic bone quality and the complexity of distal femoral periprosthetic fractures, partial weight‐bearing was typically initiated at approximately 6 weeks postoperatively after radiographic confirmation of maintained alignment and fixation stability (Figure 4). Progressive advancement to full weight‐bearing was permitted by 12 weeks after surgery in all patients, contingent upon clinical tolerance and radiographic evidence of fracture healing.

**FIGURE 4:**
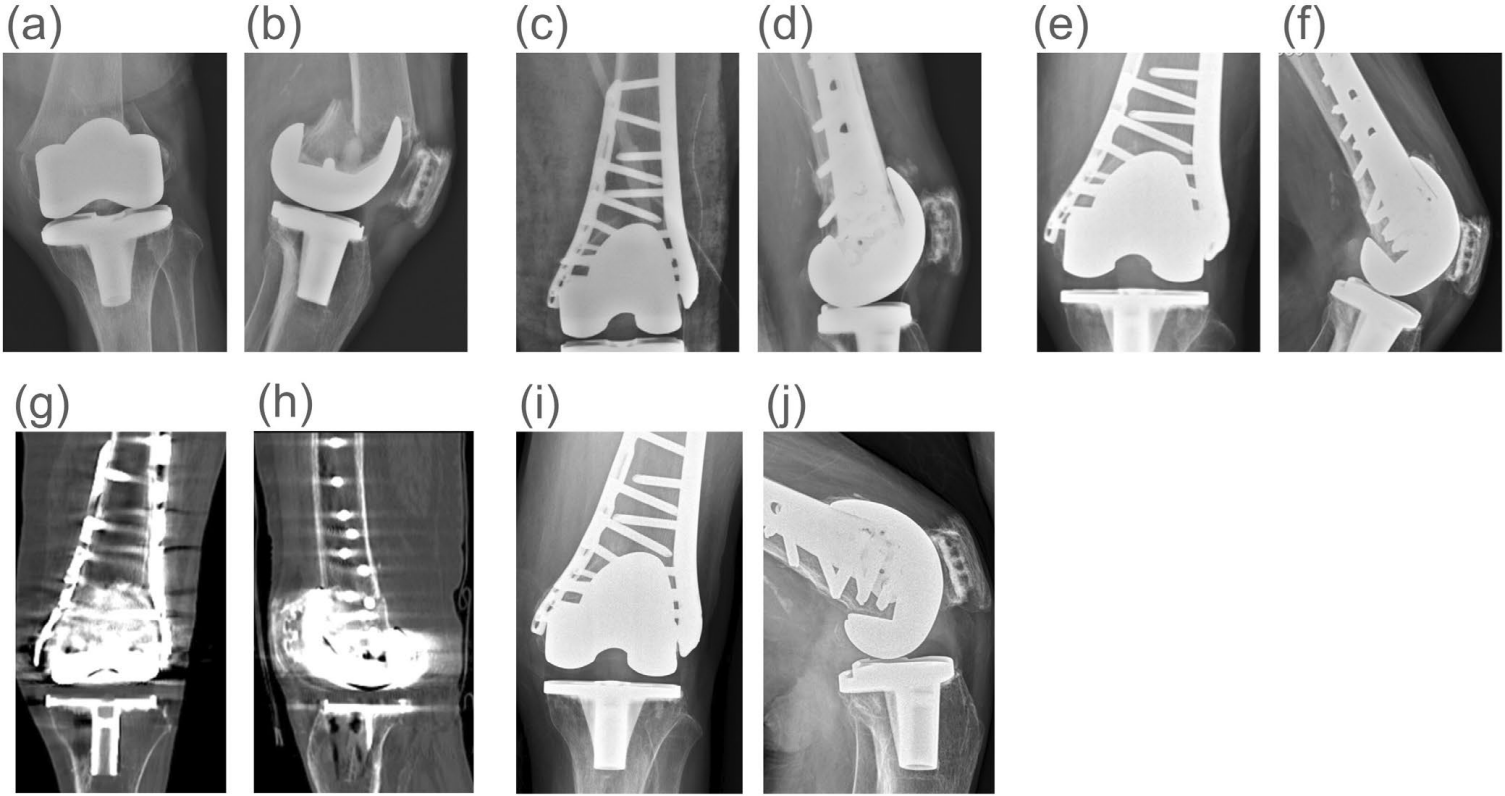
An 89‐year‐old woman with a left distal femoral periprosthetic fracture after a fall. (a) and (b) Preoperative radiographs obtained in the emergency department. (c) and (d) Postoperative radiographs after dual‐plate fixation. (e) and (f) Radiographic bony union at 3 months postoperatively. (g) and (h) CT confirmation of bony union at 6 months postoperatively. (i) and (j) Latest radiographic image at 18 months postoperatively.

## 3 Results

### 3.1 Patient Characteristics and Perioperative Data

Patient characteristics and perioperative data are summarized in Table 2. All 10 patients were women, with a mean age of 80 years (range, 63–89 years). Four patients sustained right‐sided fractures, and six sustained left‐sided fractures. The interval from primary total knee arthroplasty to fracture occurrence ranged from 4 to 13 years (mean, 9.4 years).

Medical comorbidities were prevalent: eight patients had at least one internal medicine‐related condition, including hypertension, diabetes mellitus, coronary artery disease, hyperlipidemia, gout, previous stroke, dementia, or end‐stage renal disease. Two patients had no known comorbidities. Operative time ranged from 146 to 215 min (mean, 172 min), and estimated intraoperative blood loss ranged from 500 to 1000 mL (mean, 760 mL). All patients required intraoperative blood transfusion (mean, 700 mL).

### 3.2 Postoperative Clinical and Radiographic Follow‐Up

All patients were followed for a minimum of 12 months, with a maximum of 18 months. Postoperative radiographs and computed tomography were used to monitor fracture healing. Radiographic bridging callus formation was observed between 4 and 6 weeks postoperatively, and bony union was achieved between 3 and 5 months in most patients (Figure 4). One patient with severe osteoporosis demonstrated slightly delayed union at 5 months. No cases of implant loosening or fixation failure were observed. Bone mineral density assessment was available for all patients, with T‐scores ranging from −2.8 to −4.0 (mean, −3.46), consistent with severe osteoporosis.

At 3 months postoperatively, most patients had regained near‐full knee ROM (0°–120°), whereas two patients demonstrated a ROM of 0°–90° at that time point. Partial weight‐bearing was initiated between 6 and 8 weeks postoperatively and progressed to full weight‐bearing by 12–14 weeks in all patients. At 1‐year follow‐up, the mean KOOS was 89 (range, 81–95), and the mean VAS pain score was 1.7 (range, 1–3), reflecting satisfactory functional recovery and minimal residual pain. Subscale analysis demonstrated consistently favorable outcomes across pain, symptoms, activities of daily living, sports/recreation, and quality‐of‐life domains.

Minor transient complications included mild local swelling in two patients and superficial wound erythema in one patient, which resolved with conservative management. No deep infections, thromboembolic events, or revision surgeries were required.

Collectively, these results suggest that dual‐plating ORIF using lateral LISS and medial distal tibial metaphyseal plates can provide reliable fracture union, stable fixation, and favorable mid‐term functional outcomes in elderly patients with compromised bone quality. Given the small sample size and retrospective design, these findings should be considered preliminary and hypothesis‐generating.

## 4 Discussion

According to data collected from patients' clinical and functional outcomes, medial distal tibial metaphyseal plates not only provide reliable fracture union, stable fixation, and favorable mid‐term functional outcomes in elderly patients with compromised bone quality, but also have the benefits of lower implant profile, curvature fitness without additional intra‐operative bending.

### 4.1 Biomechanics of Lateral Plating Only and Dual‐Plate

Traditionally, the AO Less Invasive Stabilization System (LISS) locking plate has been the primary implant for extra‐articular distal femoral fractures. However, with the increasing incidence of comminuted distal femoral and periprosthetic fractures, dual‐plating constructs have gained popularity, adopting biomechanical principles analogous to the “triangle of stability” concept established in distal humerus fixation [24, 25]. Clinical and biomechanical studies suggest that dual‐plating constructs may provide superior stability and higher union rates compared with lateral single‐plate fixation, particularly in osteoporotic bone [7, 16, 27]. Lateral‐only plating, by contrast, has been associated with nonunion and implant failure rates approaching 20% in select cohorts [20], highlighting the potential limitations of unilateral support.

### 4.2 Postoperative Outcomes of Dual‐Plating Fixation

In distal femoral periprosthetic fractures, dual plating provides a critical biomechanical advantage: medial buttress support [17]. The medial column counteracts varus forces generated by the physiologic mechanical axis of the lower limb. Without medial support, lateral‐only constructs are vulnerable to progressive varus collapse and fixation failure [19, 21]. In our series, dual plating allowed patients to begin partial weight‐bearing at 6–8 weeks postoperatively without loss of alignment or construct compromise, consistent with recent reports of improved knee range of motion, higher union rates, and favorable patient‐reported outcomes compared with single‐plate fixation [7, 16, 27].

### 4.3 Surgical Approaches for Dual‐Plating Fixation

Various surgical approaches for dual‐plating fixation have been described [28]. While some authors have reported satisfactory outcomes using a single extensile medial or lateral parapatellar approach [10], we adopted separate medial and lateral incisions through intermuscular planes. This approach provided superior visualization of both columns, facilitated accurate anatomic reduction, and enabled precise implant positioning without substantially increasing operative time. We believe this method is particularly advantageous in complex periprosthetic fracture patterns where precise reduction is essential to restore limb alignment and construct stability.

### 4.4 Implant Selection for Medial Side of Dual‐Plating Fixation

Implant selection for medial augmentation remains debated. Prior studies, such as Park et al., used PHILOS plates for medial support via a minimally invasive technique [23]. However, the PHILOS plate was designed for proximal humerus anatomy and may require intraoperative contouring to fit the distal femur. In contrast, the DePuy distal tibial metaphyseal plate conforms closely to the medial curvature of the distal femur, provides multiple fixation points with versatile screw trajectories, and maintains intimate plate‐to‐bone contact. This is particularly valuable in osteoporotic bone and in the presence of a femoral component, where screw placement options may be limited. The plate's low‐profile design may also minimize soft tissue irritation and improve postoperative knee range of motion. Collectively, these features offer biomechanical advantages and facilitate reliable medial buttress support until a dedicated medial distal femoral plate becomes available.

### 4.5 Assessment of Patients Indicated for Dual‐Plating Fixation of Fracture

A critical consideration in periprosthetic fracture management is femoral component stability. All patients in this series underwent careful preoperative radiographic assessment and intraoperative verification of a well‐fixed femoral component. In cases where prosthetic loosening is suspected, dual plating alone would be insufficient, and revision arthroplasty with a long‐stem component may be indicated. By explicitly confirming component stability, we ensured that our results reflect the outcomes of dual‐plating fixation in the context of an intact prosthesis.

## 5 Limitations and Strengths

Several limitations must be acknowledged. First, the retrospective design and small sample size limit the strength of the evidence and preclude definitive conclusions regarding the superiority of this fixation strategy. Second, distal femoral periprosthetic fractures are relatively uncommon and often demonstrate substantial heterogeneity in fracture configuration, bone quality, prosthesis design, and fixation requirements, making the accumulation of a large, homogeneous cohort challenging. Third, the present study was designed as a preliminary case series to demonstrate the technical feasibility and clinical applicability of using a distal tibial metaphyseal plate as a medial distal femoral plate, rather than to establish comparative efficacy. Moreover, all the patients in our study were females. Gender is one of the variables that could lead to bias in the final result, since osteoporosis is much more common in females than males [29]. These results should therefore be interpreted as preliminary and hypothesis‐generating. Future studies with larger, multicenter cohorts and longer follow‐up are warranted to validate the biomechanical and clinical benefits of this construct.

To our knowledge, this study is the first to report routine use of a medial distal tibial metaphyseal plate for fixation on the medial side of the distal femur, conforming to the current concept of a dual‐plating technique combining a lateral LISS plate, with more fitness of the curvature and no additional intra‐operative bending. Although there is currently no available medial distal femoral anatomical plate, our idea with the utilization of a medial distal tibial metaphyseal plate on the medial femur demonstrates rigid fixation and a lower implant profile that allowed early mobilization, high union rates, and satisfactory functional outcomes without major mechanical or wound complications.

The main purpose of this study is to provide another thinking process that emphasizes fracture fixation should not only be limited to the frame of anatomical plate, nor completely comparing the postoperative outcome with other surgical techniques or methods. Despite the small sample size of only 10 cases and retrospective design in our study, we still want to convey the concept that flexible utilization of preexisting implant will undoubtedly achieve the best fracture fixation effect and clinical outcome.

## 6 Conclusion

Using medial distal tibial metaphyseal plate for medial augmentation represents a reliable surgical strategy for managing distal femoral periprosthetic fractures after TKA, providing further enhanced construct stability, enabling early mobilization, favorable anatomic conformity, effective medial buttress support, and secure fixation in osteoporotic bone. In this series, we deliver the concept that the principles of fracture fixation should not be limited by the designation of a given implant as an anatomical plate. Instead, surgeons should consider the fracture morphology, local anatomy, and biomechanical requirements, and adapt the available implants to achieve the most appropriate and stable construct for each individual case. While preliminary, these results support the potential utility of preexisting implant and provide a foundation for further investigation in larger, long‐term studies. Further prospective comparative studies with larger cohorts are required to validate the clinical effectiveness, safety, and reproducibility of this technique.

## Author Contributions

**Che Lin:** writing – original draft, conceptualization, methodology, data curation, formal analysis, investigation. **Yueh‐Ying Hsieh:** writing – review and editing, supervision, project administration, validation, visualization.

## Funding

The authors have nothing to report.

## Ethics Statement

The study protocol was reviewed and approved by the Institutional Ethics Committee (Approval No. N202604129). All procedures were conducted in accordance with the principles of the Declaration of Helsinki.

## Consent

The requirement for individual informed consent was waived by the Institutional Ethics Committee because this study involved retrospective analysis of anonymized clinical data.

## Conflicts of Interest

The authors declare no conflicts of interest.

## Data Availability

The data that support the findings of this study are available on request from the corresponding author. The data are not publicly available due to privacy or ethical restrictions.
